# Lateralization of inferior petrosal sinus sampling in Cushing's disease correlates with cavernous sinus venous drainage patterns, but not tumor lateralization

**DOI:** 10.1016/j.heliyon.2020.e05299

**Published:** 2020-10-22

**Authors:** Mohammad Ghorbani, Hamideh Akbari, Christoph J. Griessenauer, Christoph Wipplinger, Alireza Dastmalchi, Mojtaba Malek, Iraj Heydari, Reza Mollahoseini, Mohammad E. Khamseh

**Affiliations:** a Division of Vascular and Endovascular Neurosurgery, Firoozgar Hospital, Iran University of Medical Sciences, Tehran, Iran; b Clinical Research Development Unit, Sayad Shirazi Hospital, Golestan University of Medical Sciences, Gorgan, Iran; c Department of Neurosurgery, Geisinger Health System, Danville, PA; d Research Institute of Neurointervention, Paracelsus Medical University, Salzburg, Austria; e Department of Neurosurgery, Medical University of Innsbruck, Innsbruck, Austria; f Endocrine Research Center, Institute of Endocrinology and Metabolism, Iran University of Medical Sciences (IUMS), Tehran, Iran

**Keywords:** Anatomy, Neurology, Medical imaging, Endocrinology, Endocrine system, Clinical research, Inferior petrosal sinus sampling, Cushing's disease, Parasellar venous drainage, Lateralization of microadenoma

## Abstract

**Background:**

Inferior petrosal sinus sampling (IPSS) is known as the gold standard to distinguish whether excessive adrenocorticotropin hormone (ACTH) production origins from the pituitary gland or an ectopic source. However, due to a number of factors, the value of IPSS for adenoma lateralization may be limited. Aim of this study was to evaluate the influence of parasellar venous drainage (VD) patterns on IPSS findings in predicting lateralization of pituitary microadenomas.

**Methods:**

We retrospectively reviewed records of confirmed cases of Cushing's disease which were evaluated by IPSS prior to endoscopic tansnasal trans-sphenoidal surgery (ETSS) to assess the ability of IPSS to predict adenoma laterality.

**Results:**

Seventeen patients with pathologically confirmed Cushing's disease were retrospectively reviewed. The median age of the included patients was 37 years. Laterality of parasellar VD perfectly associated with lateralization as measured by IPSS. Symmetrical VD was associated with symmetrical ACTH gradient on IPSS. However, lateralization measured by IPSS did not show any significant correlation with lateralization detected during ETSS.

**Conclusion:**

Our study suggests that IPSS lateralization results strongly depend on parasellar VD pattern but show no significant correlation with the adenoma lateralization found during ETSS. Thus, IPSS does not appear to be an appropriate modality to predict adenoma lateralization.

## 1 Introduction

Cushing's disease results from an adrenocorticotropin hormone (ACTH) secreting pituitary adenoma [1]. The term adenoma lateralization refers to the anatomical location of the adenoma within the pituitary gland. Gold standard for confirming Cushing's disease is inferior petrosal sinus sampling (IPSS). Higher ACTH concentrations in one or both inferior petrosal sinuses (IPS) compared to peripheral blood samples is diagnostic for Cushing's disease [2,3].

IPSS was described to have a sensitivity and specificity of 100% [4,5]. It has been hypothesized that in 78% of patients with Cushing's disease an intergradient difference between the two IPS of 1.4 to 1 and above can accurately predict the site of the adenoma (right, left, or middle of the pituitary gland). This is referred to as IPSS lateralization [6, 7, 8]. However, several studies indicated that parasellar venous anatomical variations potentially lead to failure in correctly predicting adenoma lateralization via IPSS [9,10]. Particularly in very small microadenomas it is most perilous to rely on IPSS results for choosing which side of the pituitary gland to resect [11]. We hypothesized that IPSS lateralization is dependent on the parasellar venous drainage (VD) pattern rather than the tumor location. Therefore, we aimed to design a retrospective study to evaluate the effect of parasellar VD pattern on IPSS.

## 2 Material and methods

We retrospectively reviewed records of definite cases of Cushing's disease that were evaluated by IPSS followed by endoscopic transsphenoidal surgery (ETSS) in our institutional database between January 2014 and October 2018. All patients were evaluated by an expert team including interventional neurosurgeons and neuroendocrinologists to avoid patient selection bias. Our research protocol was approved by the local ethics committee. We collected demographic data including age, sex, ETSS reports, preoperative dynamic contrast sella MRI (dMRI), IPSS, and VD pattern results.

### 2.1 IPSS procedure

**Figure 1:**
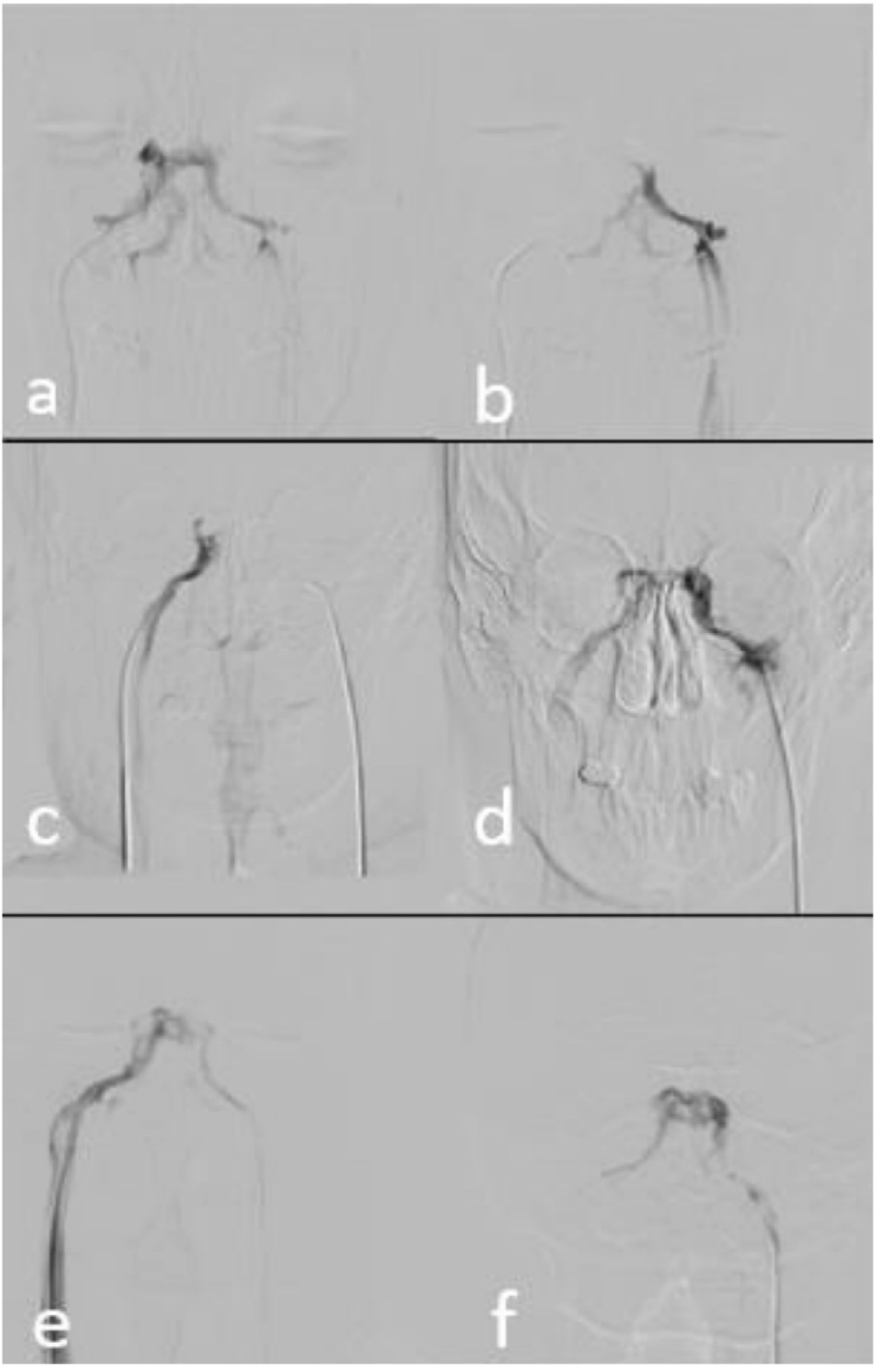
Superselective venography of bilateral inferior petrosal sinuses (IPS). (a & b) Left-sided dominance of venous drainage pattern is evident when right-sided injected contrast passes thorough the midline and fills the contralateral cavernous sinus (CS) and IPS while left-sided injection fills only the left side due to the direction of venous flow. (c & d) Right-sided dominance with a reverse pattern of parasellar venous drainage toward right CS and IPS. (e & f) Bilateral superselective venography shows symmetric pattern of parasellar venous drainage passing through the midline with bilateral injections.

The patient was placed in supine position on the fluoroscopy table and both groins were sterilized with antiseptic solution. Local anesthesia with 2% Lidocaine was applied. Using Seldinger technique, two 5F and 7F sheaths were inserted bilaterally into bilateral femoral veins. Two 5F vertebral catheters were passed through the right atrium into and through the superior vena cava, into the left brachiocephalic vein while the other was advanced into the right internal jugular veins (IJV) by using a 0.035″ hydrophilic guiding wire. Both catheters were positioned in the orifice of bilateral inferior petrosal sinuses. After this, two 0.018″ microcatheters were introduced to the bilateral IPS over 0.014″ microwires. VD pattern was determined by superselective contrast injection into the right and left IPS respectively. If, upon injection, contrast filled the ipsilateral side of the cavernous sinus then passed the midline via inter-cavernous coronary veins and filled contralateral cavernous sinus and IPS, the VD pattern was considered symmetric or dominant on the contralateral side. If contrast only filled the ipsilateral IPS and cavernous sinus, the dominancy of parasellar VD was considered ipsilateral to injection (Figure 1).

Next, 10 μg DDAVP was utilized for IV injection and at the times of -3, 0, 3, 10 and 15 min, simultaneous samples from right and left IPS as well as peripherally from the 7F femoral sheath were obtained and immediately put on ice for laboratory evaluation of serum ACTH and prolactin levels within less than 15 min.

### 2.2 IPSS analysis

ACTH concentration in samples from the left and right IPS as well as the peripheral venous blood were collected simultaneously. IPS to peripheral blood ACTH ratio of >2 at baseline and IPS to peripheral blood ACTH ratio >3 post DDAVP were considered diagnostic for Cushing's disease. Lateralization of the adenoma was assumed in cases where the intergradient difference was more than 1.4 to 1.

### 2.3 Endoscopic transsphenoidal surgery: (ETSS)

**Figure 2:**
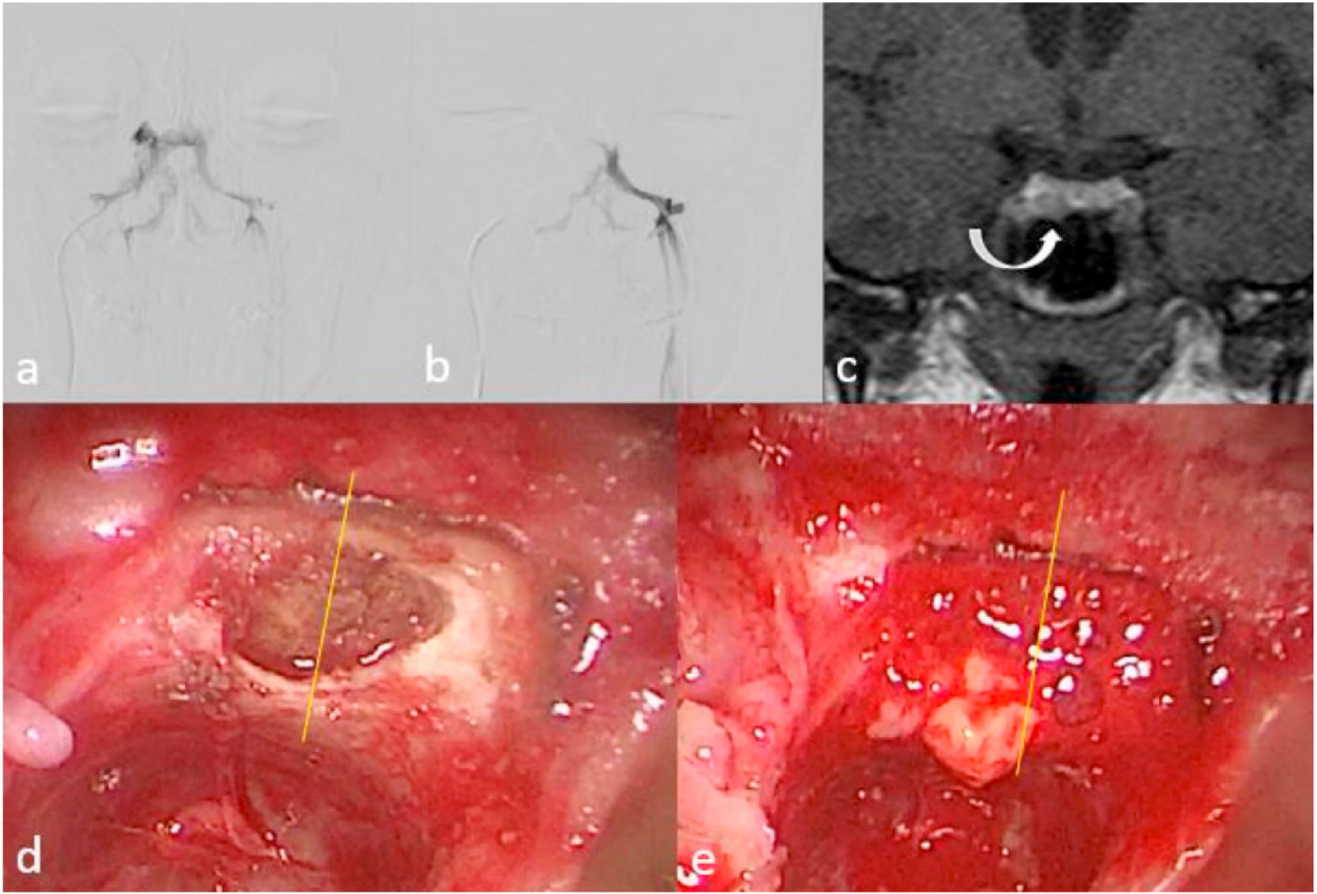
Illustration of correlation between parasellar venous drainage pattern, site of microadenoma in MRI, and during endoscopic transsphenoidal surgery. (a & b) Superselective venography of bilateral IPS shows left dominance of parasellar venous drainage pattern that is compatible with an IPSS gradient higher on the left side. (c) Dynamic pituitary MRI shows a microadenoma on the right side of pituitary gland (curved arrow). (d & e) Endoscopic transsphenoidal view of the same patient during surgery shows herniation of the microadenoma at the right side of sella after dural incision that is compatible with the MRI finding and opposite to IPSS gradient.

Microadenoma resection was performed via mononostrile ETSS including lateralization of nasal turbinates, opening of the sphenoid sinus, drilling of the sellar floor, incisional sellar dura matter opening and resection of the microadenoma under endoscopic view. Sellar floor repair was achieved by autograft fat pad and fascia lata or pediculated nasoseptal flap, if necessary. The lateralization of the adenoma was determined intraoperatively based on the epicenter of the tumor. It was defined as right or left if the epicenter was placed off the pituitary and central if the epicenter was on the midline (Figure 2).

### 2.4 Histopathological analysis

The definitive diagnosis of Cushing's disease was confirmed by histopathology. Histopathological analysis was performed by experienced neuropathologists. Specimens were analyzed using hematoxylin and reticulin stain as well as immunohistochemistry for ACTH and other pituitary hormones.

### 2.5 Statistical analysis

Descriptive statistics were performed to analyze patient and tumor characteristics. Correlation of surgical tumor location with adenoma lateralization on IPSS, VD, and brain MRI was assessed using Chi-Square. All statistical analysis was performed using SPSS (version 24 SPSS Inc., Chicago, IL, USA).

## 3 Results

We reviewed a total of 17 histopathologically confirmed cases of Cushing's disease between January 2014 and October of 2018. All of the cases met the following diagnostic criteria: baseline ACTH and cortisol, low dose and high dose dexamethasone suppression tests as well as the results of baseline and DDAVP stimulation tests (IP to peripheral blood ACTH ratio of >2 at baseline and IPS to peripheral blood ACTH ratio >3 post DDAVP were considered diagnostic for Cushing's disease). Dynamic sella MRI findings were obtained and showed pituitary microadenomas in all of 17 cases (100%). The median age was 37 years (range: 15–59) including 10 (58.8%) females and 7 (41.2%) males.

### 3.1 Lateralization using IPSS, VD pattern, and MRI of brain

The IPSS lateralization and VD pattern correlated in all patients. IPSS showed right-sided lateralization in 8 patients (47.1%), left-sided in 5 (29.4%) and symmetric in 4 (23.5%) patients. MRI identified 2 (11.8%) patients with right, 3 (17.6%) with left, 9 (52.9%) with central, 2 (11.8%) with left & central, and 1 (5.9%) with right & central adenomas (Table 1).

**Table 1:** Patient and tumor characteristics.

Variable	Number (percent)
Age (median and range in years)	37 (15–59)
Gender	
Female	10 (58.8%)
Male	7 (41.2%)
IPSS lateralization	
Right	8 (47.1%)
Left	5 (29.4%)
Symmetric	4 (23.5%)
Venous drainage pattern	
Right	8 (47.1%)
Left	5 (29.4%)
Symmetric	4 (23.5%)
dMRI lateralization	
Right	2 (11.8%)
Left	3 (17.6%)
Central	9 (52.9%)
Left & central	2 (11.8%)
Right & central	1 (5.9%)
Transphenoidal surgery lateralization	
Right	3 (17.6%)
Left	1 (5.9%)
Central	9 (52.9%)
Left & central	3 (17.6%)
Right & central	1 (5.9%)

### 3.2 Correlation with intra-operative lateralization

ETSS found right-sided adenomas in 3 (17.6%), left-sided in 1 (5.9%), central in 9 (52.9%), left & central in 3 (17.6%) and right & central in 1 (5.9%) (Table 1). There was no significant correlation between intra-operative tumor location and lateralization on IPSS (p = 0.431) and VD (p = 0.431). There was, however, a significant association between tumor lateralization on dMRI and ETSS (p < 0.0001) (Table 2). Dynamic MRI, could accurately predict 86% of tumor locations. IPSS lateralization on the other hand only correlated in 25% with the actual location of adenomas.

**Table 2:** Correlation of tumor location during transphenoidal surgery and lateralization by IPSS, venous drainage, and dMRI.

	IPSS			p-value
	Right	Left	Symmetric	
Venous drainage:				
VD (right)	8 (100%)	0	0	**P = 1**
VD (left)	0	5 (100%)	0	
VD (symmetric)	0	0	4 (100%)	
MRI:				
MRI (right)	0	2 (100%)	0	**<0.0001**
MRI (left)	1 (33.3%)	1 (33.3%)	1 (33.3%)	
MRI (central)	5 (55.6%)	2 (22.2%)	2 (22.2%)	
MRI (central and left)	1 (50%)	0	1 (50%)	
MRI (central and right)	1 (100%)	0	0	
Surgery:				
Main location (right)	0	2 (66.7%)	1 (33.3%)	**0.431**
Main location (left)	0	1 (100%)	0	
Main location (central)	5 (55.6%)	2 (22.2%)	2 (22.2%)	
Main location (central and left)	2 (66.7%)	0	1 (33.3%)	
Main location (central and right)	1 (100%)	0	0	
Total	8 (47.1%)	5 (29.4%)	4 (23.5%)	

Bold value indicates correlation of tumor location during transphenoidal surgery and lateralization by IPSS, venous drainage, and brain MRI.

## 4 Discussion

In the present study, we performed superselective venography to evaluate VD pattern during IPSS. Furthermore, lateralization of the tumor was assessed by dMRI and IPSS, and compared to definitive results from tissue samples obtained during surgery. We found that the VD pattern could accurately predict IPSS lateralization in all patients. However, IPSS lateralization failed to show any significant correlation with the definite tumor location among our study population.

### 4.1 Factors diminishing predictive value of IPSS lateralization

There are studies suggesting that IPSS outcomes are influenced by factors such as blood flow pattern of the cranial venous system [12] thus leading to uncertainty of IPSS outcomes in terms of lateralization. Multiple factors including asymmetric or hypoplastic petrosal sinus anatomy [7,11,24] and asymmetric bilateral catheter positioning [11] have been postulated as potential sources of error in predicting tumor lateralization. Wind et al. explained that mechanisms such as intercavernous venous mixing or dominance in VD patterns that are not evident on venographically displayed anatomy, may also contribute to false-positive results [13].

### 4.2 Methods for detecting adenoma lateralization

Sun et al. conducted a retrospective study in 2015, aiming to evaluate the accuracy of IPSS, dMRI, and cavernous sinus sampling in 30 patients. Dynamic MRI outcomes had a significant correlation with lateralization found after surgery (r = 0.5, p < 0.002). However, the results of cavernous sinus sampling had no association with intraoperatively found lateralization of the tumor (r = 0.14, p = 0.4). Hence, the dynamic MRI could help to discover the site of tumor more accurately than CSS [14]. This is in accordance to our study results that indicated that dMRI could predict the tumor location more accurately than IPSS results. In a retrospective study published in 2003, Lefournier et al. evaluated the accuracy of modalities for localizing the tumor lateralization. Overall, in 57% of patients, adenoma lateralization could be localized accurately by IPSS. Accuracy was 86% with both catheters in inferior petrosal sinuses compared to 50% accuracy with both catheters in the cavernous sinuses. Consequently, the study suggested that IPSS is more accurate if VD is symmetric. Due to cranial nerve six palsies, occurring after cavernous sinus catheterization, this procedure may be considered unsafe [11].

### 4.3 Sensitivity of IPSS lateralization

In the present study, we revealed a low diagnostic utility of IPSS for lateralization of pituitary microadenomas when compared to the previous observations. In other words, no significant correlation was revealed between intra-operative tumor location as the gold standard and lateralization on IPSS. As previously shown by Chen et al. in 2019 [15], the sensitivity of IPSS lateralization among children was shown to be 64.7%, and up to 8.3.3% in cases where DDAVP was used. This was considerably higher than what we found in the present study. In studies of adults, the reported sensitivity was even higher than in children. The sensitivity of IPSS in detecting Cushing's disease lateralization was higher than 80% [16]. In another experience by the Peking Union Medical College Hospital (PUMCH), the sensitivity of IPSS was shown to be 90.1% without DDAVP stimulation and in the range of 95.6–98.9% after stimulation [17,18]. It seems that the criteria used for assessing the tumor lateralization accuracy in different studies may have been different. In this regard, in some studies, only patients with tumors in the midline were included in the calculation [19,20] resulting high false negative results. It should be also considered that other parameters such as the site of tumor and the number of operators might affect the sensitivity of IPSS [21]. Although radiological imaging is less sensitive than IPSS in detecting Cushing's disease, it may be superior to IPSS for diagnosing localization of the intra-pituitary site of the lesion. The discrepancy between the different studies could also be due to the mixing of blood from the two sides of the anterior pituitary [22]. It has been clearly demonstrated asymmetric drainage from cavernous sinuses to the IPSS assessed by cavernous sinus venography [23] can lead to blood draining from both cavernous sinuses into a single IPS.

### 4.4 Dynamic contrast sella MRI for lateralization

It has been shown that even with the introduction of dynamic MRI, the ability to identify pituitary tumors fails in as many as 50% of Cushing's disease patients [24,25], probably due to the very small size of the pituitary adenomas. IPSS is now applied as a standard diagnostic test in patients with suspected Cushing's disease who have either had inconclusive biochemical results or negative MRIs [26, 27, 28]. Thus, we believe that IPSS can be helpful in localizing pituitary adenomas in patients where no adenoma is identifiable on MRI. Of course, it should also be pointed out that although the sensitivity of contrast-enhanced pituitary MRI can be slightly increased by acquiring a dynamic sequence immediately after contrast injection [29,30], this technique has not unequivocally demonstrated to improve the usefulness of MRI in Cushing's disease.

Our current study demonstrated that parasellar VD pattern can be shown at the time of IPSS via superselective bilateral venography. We also demonstrated that the VD pattern is the most important influencing factor on the of ACTH gradient in IPSS. The role of VD pattern evaluated by superselective bilateral venography and its association to IPSS lateralization results was rarely assessed and discussed in previous studies. To our knowledge this is the first study to show 100% correlation of VD pattern with IPSS lateralization.

### 4.5 Limitations

Currently, Corticotropin releasing hormone (CRH) is the preferred stimulating agent in IPSS, but the drug is not available in our country. DDAVP, however, has been used as an alternative in several previous studies [31].

## 5 Conclusions

Our findings indicate that superselective venography during IPSS is helpful for the interpretation of IPSS results by evaluating the VD pattern. IPSS lateralization in Cushing's disease is dependent on the parasellar VD pattern and not on the site of microadenomas. The site of microadenomas found on MRI shows stronger correlation with definitive findings during surgery and should be considered as the main modality for determining lateralization prior to surgery.

## Declarations

### Author contribution statement

M. Ghorbani: Conceived and designed the experiments; Performed the experiments; Wrote the paper.

H, Akbari and M. Khamse: Performed the experiments.

A. Dastmalchi: Performed the experiments; Analyzed and interpreted the data; Wrote the paper.

C. Griessenauer, C. Wipplinger, M. Malek, I. Heydari and R. Mollahoseini: Contributed reagents, materials, analysis tools or data.

### Funding statement

This research did not receive any specific grant from funding agencies in the public, commercial, or not-for-profit sectors.

### Competing interest statement

The authors declare no conflict of interest.

### Additional information

No additional information is available for this paper.
